# Xpert^®^ MTB/RIF assay on formalin-fixed paraffin-embedded tissues in the diagnosis of extrapulmonary tuberculosis

**DOI:** 10.4102/ajlm.v8i1.748

**Published:** 2019-09-18

**Authors:** Allan N. Njau, Samuel M. Gakinya, Shahin Sayed, Zahir Moloo

**Affiliations:** 1 Department of Pathology and Laboratory Medicine, Aga Khan University Hospital, Nairobi, Kenya

**Keywords:** Xpert MTB/RIF, extrapulmonary tuberculosis, formalin-fixed paraffin-embedded tissues, Kenya

## Abstract

**Background:**

Diagnosis of extrapulmonary tuberculosis continues to be a challenge due to the complexity of the causative organism and the wide array of pathologic features seen in this infection. Xpert MTB/RIF can be used on fresh or frozen tissue specimens for diagnosis of tuberculosis with good results. However, there is little data on its use with formalin-fixed paraffin-embedded (FFPE) tissues.

**Objectives:**

The aim of this study was to demonstrate the potential utility of Xpert MTB/RIF and to compare its performance to Ziehl-Neelsen staining for the detection of *Mycobacterium tuberculosis* from FFPE tissues using histological features from haematoxylin and eosin staining as the gold standard.

**Methods:**

Eighty randomly selected archival FFPE tissues exhibiting histological features of tuberculosis were included in the study. After deparaffinisation and lysis, all the tissue specimens were subjected to the Xpert^®^ MTB/RIF assay. The outcome measures were proportions of positively identified cases by each test.

**Results:**

Using histology as the gold standard, the sensitivity of Ziehl-Neelsen staining was 20.3% (95% confidence interval: 12% – 30.8%), and the sensitivity of the Xpert^®^ MTB/RIF assay was 53.2% (95% confidence interval: 41.6% – 64.9%); the difference was statistically significant (*p* = 0.002). None of the cases tested positive for rifampicin resistance.

**Conclusion:**

With prior deparaffinisation and lysis, FFPE tissues are amenable to testing by Xpert^®^ MTB/RIF assay. A validation study to determine the clinical utility, analytical optimisation and cost implications of this assay for FFPE tissues is recommended.

## Introduction

Globally, extrapulmonary tuberculosis (EPTB) represented 15% of the 6.3 million cases and in Africa 16% of the 1.2 million cases that were notified in 2016. Children and the immunosuppressed population have been reported to be at a higher risk of EPTB than the general population.^1,2^

Diagnosis of EPTB involves analysis of fresh or fixed tissue biopsies using culture, histological examination or molecular tests. Culture is regarded as the gold standard; however, its main disadvantages are the long turnaround time – up to 6 weeks – and the fact that only fresh tissue can be used. In addition, the sensitivity of cultures has been found to be variable (0% – 80%).^3^ Histological diagnosis of tuberculosis on haematoxylin and eosin (H&E) stained sections usually relies on the presence of classical necrotising granulomas with Langhans giant cells. However, tuberculosis can exhibit other patterns of inflammation such as non-necrotising granulomatous inflammation, non-specific chronic inflammatory processes and suppurative inflammation.^3,4,5^ In HIV and tuberculosis co-infection for example, there may be both delay and poor or no formation of granulomas.^6^ In addition, other agents and pathologic conditions can mimic tuberculosis. Examples include infections caused by nontuberculous mycobacteria, bacteria other than mycobacteria, fungi, some viruses and parasites as well as non-infective causes such as Vasculitides and Crohn’s disease.^7,8^ Presence of acid-fast bacilli (AFB) on Ziehl-Neelsen (ZN) staining is widely utilised as a confirmatory test for EPTB in a wide spectrum of formalin fixed paraffin embedded (FFPE) tissues because it is quick and relatively cheap; however, its drawback is its low sensitivity (40%).^3,9,10^

Both commercial and in-house polymerase chain reaction (PCR) based assays for tuberculosis have been used to improve detection rates of EPTB in FFPE tissues and to overcome some of the limitations encountered by the aforementioned tests.^11,12^ Polymerase chain reaction based assays have been found to be more powerful in ruling in rather than ruling out a diagnosis of tuberculosis.^10^ A major reason for this is the fact that most EPTB tissue specimens tend to be paucibacillary. Moreover, the bacilli are usually not uniformly distributed throughout the specimens.^12,13,14^

The Xpert^®^ MTB/RIF assay (Cepheid, Sunnyvale, California, United States) is an automated semi-nested real time PCR in which sample lysis, DNA extraction, amplification and detection takes place within a single plastic cartridge. The assay detects the presence of the *M. tuberculosis* complex and rifampicin resistance within 2 hours.^15,16^ The World Health Organization initially endorsed the assay in 2010 as a rapid nucleic acid amplification assay that simultaneously detects the presence of the *M. tuberculosis* complex and rifampicin resistance in sputa. However, a wide spectrum of tissues are now being analysed using the Xpert^®^ MTB/RIF assay.^17,18^ Meta-analysis done on the accuracy of Xpert MTB/RIF in detecting tuberculosis shows that the median pooled sensitivity and specificity in lymph nodes was 84.9% and 92.5%, while in tissues other than lymph nodes, the median pooled sensitivity and specificity was 81.2% and 98.1%.^19^ In these studies, the nature of the specimens was reported to be either fresh or frozen. A Zambian study published in 2017 by Polepole et al. showed Xpert^®^ MTB/RIF assay to be potentially useful for the diagnosis of tuberculosis in FFPE tissues. However, the sensitivity was found to be too low. Using histology as the gold standard, the accuracy of ZN, Xpert MTB/RIF, and in-house PCR were assessed in 100 FFPE tissues. The sensitivity of Xpert MTB/RIF within lymph nodes and non-lymph node tissues was 30% and 35%.^20^

When dealing with FFPE tissues as the starting specimen on PCR based assays, pretreatment procedures for deparaffinisation and mitigation of the effects of PCR inhibitors is necessary.^21,22,23,24^ On the other hand, only minimal bio-safety requirements are needed for FFPE tissues since they do not contain live bacilli. The Xpert MTB/RIF platform, when compared to other PCR platforms, offers additional benefits that include ease of conducting the test and interpreting the results. In addition, only minimal time is required to train laboratory staff who can operate the system.^16,18^ The Xpert MTB/RIF technology is also widely available and accessible as opposed to the other technologies that tend to be found mainly in reference laboratories.

On many occasions, tissues are submitted to the laboratory while fixed in formalin and with no possibility of culture. The low sensitivity of ZN often results in negative ZN staining. Not infrequently, tissues also lack the typical histologic features of tuberculosis. In these situations, Xpert MTB/RIF can be a useful adjunct or ‘add-on’ test for EPTB.

## Methods

### Ethical considerations

The Health Research Ethics Committee, Aga Khan University, provided approval for the study [2014/REC-56(v3)].

### Study design and setting

This was a retrospective cross-sectional laboratory based study conducted at the Aga Khan University Hospital, Nairobi, in the department of Pathology and Laboratory Medicine. The laboratory receives samples from the university hospital as well as from other county and mission hospitals within the country. Only 17% of the samples analysed in this study were from the university hospital and these were fixed in 10% neutral buffered formalin for 6–12 h prior to processing. The remaining 83% were from the other hospitals and the dilution of formalin used and duration of fixation could not be determined. The patient’s biodata, site of biopsy and relevant clinical history, when provided, were obtained from the laboratory specimen inventory and information system.

### Sampling

The study included specimens received between January and December 2014 and with a diagnosis of EPTB based on the original histology features from H&E staining (necrotising granulomatous inflammation, non-necrotising granulomatous inflammation, chronic inflammation, necrotising inflammation or suppurative inflammation). The specimens were included irrespective of their ZN results; however, specimens in which a diagnosis of EPTB had been ruled out, such as cases of fungal infection, were not considered. Out of the 132 archived FFPE tissue blocks that fit the above description, 17 were excluded because the amount of tissue preserved was too small to produce an adequate sample for H&E staining, ZN staining and the Xpert^®^ MTB/RIF assay. Out of the 115 remaining blocks, 80 FFPE blocks were randomly selected for analysis.

### Laboratory procedures

From each FFPE block, H&E and ZN stained slides were prepared on 3 *µ*m thick sections. The histological pattern of inflammation for each case was recorded. The ZN stained slides were screened for AFB under oil at ×1000 magnification and the results recorded as either positive or negative. The principal investigator first screened all the slides and then every fifth case had the H&E and ZN stained slide screened by a second pathologist. For the Xpert^®^ MTB/RIF assay, up to 10 sections of 10 *µ*m thickness were cut from the FFPE blocks and the scrolls placed in a 1.5 mL micro-centrifuge tube. Deparaffinisation was done using a modified xylene method, which involved two washes in 1 mL of xylene, two washes in 1 mL of absolute ethanol followed by two rinses in 1 mL of phosphate buffered saline. Lysis of the tissue fragments followed with resuspension of the tissue fragments in 1 mL of phosphate buffered saline and the addition of 20 *µ*L of QIAGEN proteinase K, then the mixture was incubated at 56 °C for 12 hours. If there were tissue fragments visible on inspection after the initial incubation, another 20 *µ*L of proteinase K was added and re-incubated for 1 or 2 h. After complete lysis was achieved, the proteinase K was deactivated by increasing the temperature to 94 °C for 10 min.^25^ The Xpert^®^ MTB/RIF assay was then performed following the manufacturer’s instructions using the lysate and without any further attempt to extract DNA from the lysate. As a quality control measure, the microtome blade was changed and the microtome overlay cleaned with absolute alcohol after each case to prevent carryover contamination.^6,20,21^

Xpert MTB/RIF results were recorded as either detected, not detected or error. One case resulted in an Xpert^®^ MTB/RIF assay error. The bacilli load in each specimen was also recorded following the assay’s semi-quantitative estimate of the bacilli load as either high, medium, low or very low. Rifampicin resistance was recorded as either detected, not detected or indeterminate.^15^

### Data analysis

The data were analysed using the Statistical Package for Social Sciences version 19, (IBM Corp, Armonk, New York, United States). Sensitivities were calculated from proportions of cases positive on Xpert MTB/RIF and those positively detected by ZN against histology as the gold standard. A Chi-square test was applied to test for statistical differences and *p* values less than 0.05 were considered statistically significant.

## Results

Only 79 cases were analysed after the single case without Xpert MTB/RIF results was excluded from further analysis. The mean age of patients was 27 years with an age range of 6 to 85 years. Forty-four (55.7%) specimens were from female patients, and 35 (44.3%) were from male patients. Twenty-six different types of tissues were analysed and classified into nine groups according to the topographic site of the biopsy (Table 1). Lymph nodes were the most common site, 30 specimens (38%), followed by the female genital tract, 15 specimens (19%), and the abdominal cavity, 11 specimens (13.9%). The least prevalent sites were the male genital tract and the central nervous system with only one specimen each. Clinical information was available for 48.1% (38/79) of the specimens, of which 13.2% (5/38) were reported to have confirmed tuberculosis in a site other than the tissue submitted. Xpert MTB/RIF was positive for all five specimens, and ZN was positive for three of the five specimens. In 36.8% (14/38) of the specimens with clinical information, tuberculosis was clinically suspected; 5 out of the 14 were positive by Xpert MTB/RIF and 1 by ZN. HIV infection was reported in six (15.8%) of the specimens, of which four were positive by Xpert MTB/RIF and two by ZN.

**TABLE 1:** Demographic and topographic sites of tissues.

Characteristic	Value (*n* = 79)	
	*n*	%
**Age**		
Mean (range)	27 (6–85)	-
**Sex**		
Male	35	44.3
Female	44	55.7
**Topographic sites of tissues**		
Lymph nodes	30	38.0
Female genital tract	15	19.0
Abdominal tissues	11	13.9
Soft tissues	9	11.4
Joint tissues	6	7.6
Pleura and pericardium	4	5.1
Bone	2	2.5
Central nervous system	1	1.3
Male genital tract	1	1.3

Necrotising granulomatous inflammation was the predominant histological pattern of inflammation contributing to 91.1% (72/79) of the specimens (Table 2). In this group, 53% (38/72) were positive by Xpert MTB/RIF and 22% (16/72) by ZN. The contribution of granulomatous inflammation, chronic inflammation, necrotising inflammation and suppurative inflammation was marginal. In this latter group of inflammation combined, 57% (4/7) were positive by Xpert MTB/RIF and none by ZN.

**TABLE 2:** Yield of Ziehl-Neelsen and Xpert MTB/RIF by patterns of inflammation.

Histologic pattern	Ziehl-Neelsen staining				Xpert MTB/RIF			
	Negative		Positive		Negative		Positive	
	*n*	%	*N*	%	*N*	%	*n*	%
Necrotising granulomatous inflammation (*n* = 72)	56	78	16	22	34	47	38	53
Others (*n* = 7)†	7	100	0	-	3	43	4	57

† , Granulomatous, *n* = 3; Chronic, *n* = 2; Necrotising, *n* = 1; Suppurative, *n* = 1.

In lymph node tissues, 16 out of 30 were positive by Xpert MTB/RIF compared to 8 by ZN, whereas in non-lymph node tissues, 26 of 49 were positive by Xpert MTB/RIF compared to 8 by ZN (Table 3). Xpert MTB/RIF was positive in 87.5% (14/16) of the specimens that were ZN positive. Two specimens that were ZN-positive gave a negative Xpert MTB/RIF result. Using H&E histology results as the gold standard, the overall sensitivity of ZN was 20.3% (95% confidence interval [CI]: 12% – 30.8%) and that of Xpert MTB/RIF was 53.2% (95% CI: 41.6% – 64.9%), significantly better than ZN (*p* = 0.002).

**TABLE 3:** Yield of Ziehl-Neelsen and Xpert MTB/RIF test by tissue type.

Tissue *t*	Ziehl-Neelsen staining			Xpert MTB/RIF		
	Negative	Positive	Sensitivity (CI)	Negative	Positive	Sensitivity (CI)
Lymph node tissues	22	8	26.7 (13–46.2)	14	16	53.3 (34.6–71.2)
Non-lymph node tissues	41	8	16.3 (7.8–30.2)	23	26	53.1 (38.4–67.2)
Overall sensitivity	-	-	20.3 (12–30.8)	-	-	53.2 (41.6–64.9)

CI, confidence interval.

Of the 42 Xpert MTB/RIF positive specimens, the bacilli load in 26 (61.9%) was quantified as ‘very low’, 11 (26.2%) as ‘low’ and only 5 (11.9%) as ‘medium’. None of the cases showed a ‘high’ bacilli load and neither was there any case of rifampicin resistance detected. However, 7 of the 42 Xpert MTB/RIF-positive cases showed an ‘indeterminate’ rifampicin resistance, all of which had a ‘very low’ bacilli load.

## Discussion

In this study, we compared the Xpert MTB/RIF assay to histology for the detection of *M. tuberculosis* from archived FFPE tissues. The results obtained show that Xpert MTB/RIF had a higher sensitivity (53.2%) than ZN (20.3%). Similar to other studies, we found that ZN had a low sensitivity for tuberculosis in FFPE tissues.^3^ The sensitivity of Xpert MTB/RIF in our study was comparable to, though slightly higher (53.2% vs 50%) than, a previous study, in which the performance of the QIAGEN *artus* M. tuberculosis RG PCR assay was compared to Xpert MTB/RIF among 40 FFPE tissues with histopathological features consistent with tuberculosis.^26^ Similarly, the sensitivity of Xpert MTB/RIF in our study for lymph node and non-lymph node specimens (53.1% and 53.1%) was higher than that found by Polepole et al. (30% and 35%).^20^ In further support of the usefulness of Xpert MTB/RIF, its performance was found to be better than ZN in specimens from patients reported to have tuberculosis in other sites and HIV infection.

In this study, we could not determine the specificity of Xpert MTB/RIF, because we only included tissues with histological features of tuberculosis. The two cases that were ZN positive and Xpert MTB/RIF negative may represent false negatives or may have been cases of nontuberculous mycobateria. We could however not confirm the true status, because culture was not performed. Another possible explanation is that cases of EPTB tend to be paucibacillary. Moreover, the bacilli may not be evenly distributed within the tissue,^3^ raising the possibility that the sample came from a part of the tissue that may have not harboured any organisms. Multiple (up to 10 sections, 10 *µ*m thick) sections of tissue were processed for Xpert MTB/RIF testing with the aim of increasing the chances of obtaining sections with the bacilli; however, we do not know to what extent this mitigated the above factors.

The quality of DNA is also likely to have been reduced due to the untoward effects of formalin fixation.^22^ A study by Jonathan et al. conducted to investigate the effects of fixatives and fixation times on PCR showed that formalin-fixed tissues gave reliable PCR results, if the tissue was fixed for less than 48 h.^23^ In this study, standardisation of fixation times and dilution of formalin used could not be determined for 83% of the specimens that had been referred from other hospitals in different parts of the country.

A semi-quantitative estimate of bacilli load based on the cycle threshold value is another parameter that is obtained from the Xpert^®^ MTB/RIF assay. In this study, we found that more than three-quarters of the cases (61.9% and 26.2%) had a quantification of either ‘very low’ or ‘low’. This finding is probably due to the above-mentioned pre-analytical factors: the paucibacillary nature of EPTB samples and formalin fixation.

The secondary objective of our study was to determine the prevalence of rifampicin resistance. In this study, no case of rifampicin resistance was detected. Indeterminate rifampicin resistance results were however obtained for seven of the 42 cases positive on Xpert MTB/RIF. Not surprisingly, all had a very low bacilli load. Therefore, only in the 35 cases with rifampicin resistance results could we truly say that there was no case of rifampicin resistance detected. In the study by Polepole et al., a similar problem of a large number of ‘indeterminate’ results for rifampicin resistance was also encountered. Therefore, a very low bacilli load as quantified by Xpert MTB/RIF, which subsequently limits the assessment of rifampicin resistance, may have led to this finding. It is also likely that this finding was due to the small sample size of this study.

### Limitations

This study was limited by its small sample size and its retrospective nature. The duration of fixation and the strength of the formalin used were not known for the majority of the specimens. These are important factors, because they affect the quality of the DNA extracted. Lack of culture results and additional clinical information are additional limitations, because they would have aided in the determination of the clinical utility of this assay in the context of FFPE tissues.

### Conclusion

Histological assessment of H&E and ZN stained slides forms a readily accessible and affordable diagnostic tool for EPTB. However, the inability to make a definitive diagnosis of EPTB is common due to its low sensitivity. In our study, we found that culture for EPTB was rarely done and all the samples we analysed were received in the laboratory already fixed in formalin, thereby precluding culture as a diagnostic test. Since the Xpert MTB/RIF assay is widely available in many parts of Kenya, it may potentially be an excellent ‘add-on’ or adjunct test for the detection of *M. tuberculosis* in fixed tissues. This is especially true in cases that are ZN-negative or those that lack the typical histologic features of tuberculosis. We recommend a validation study to determine the clinical utility, analytical optimisation and cost implications of this assay.
